# Silent Counterattack: The Impact of Workplace Bullying on Employee Silence

**DOI:** 10.3389/fpsyg.2020.572236

**Published:** 2020-11-23

**Authors:** Xiwei Liu, Shenggang Yang, Zhu Yao

**Affiliations:** ^1^College of Finance and Statistics, Hunan University, Changsha, China; ^2^School of Economics and Management, Tongji University, Shanghai, China

**Keywords:** workplace bullying, psychological safety, affective commitment, employee silence, forgiveness climate

## Abstract

The purpose of this paper is to explore the relationship between workplace bullying (WB) and employee silence (ES) as well as its mechanism. This paper collects data from 322 employees of three Chinese enterprises in two waves, with a 2 months interval between the two waves. Moreover, this paper uses confirmatory factor analysis, a bootstrapping mediation test, a simple slope test, and other methods to verify the hypothesis. We find that: (1) WB is positively correlated with ES; (2) psychological safety (PS) and affective commitment mediated the relationship between WB and ES, respectively, and these two variables have a chain mediating effect in the above relationship; and (3) a forgiveness climate moderates this chain mediating effect by weakening the negative impact of WB on PS. Our findings can effectively guide organizations to ultimately adjust their management style, pay attention to employees’ cognitive and emotional resources, and formulate some measures to curb WB in organizations.

## Introduction

With increasingly fierce competition prevalent in the external environment, enterprises pay more attention to employee voices since such behavior can promote their competitive advantages. As in the micro foundation of enterprises, employees are more likely to experience the deficiencies of managers’ orders and find existing problems in corporations (Benevene, 2020; Breevaart et al., 2020). However, many employees choose to keep silent and do not give feedback on the problems they find due to various factors (Morrison and Milliken, 2000; Whiteside and Barclay, 2013; Prouska and Psychogios, 2018). Such behavior is called employee silence (ES), which may pose a huge negative effect on the organization. From the perspective of employees, they can hardly perceive their value in the organization, and this thereby leads to adverse impacts such as job burnout and turnover (Morrison and Milliken, 2000; Prouska and Psychogios, 2018). Regarding managers, they are unable to gain important feedback for decision-making in a timely manner, which may result in decision bias and thus they miss the opportunity for innovation (Prouska and Psychogios, 2018; Knoll et al., 2019). Therefore, how to reduce ES behavior has attracted wide academic and practical attention.

The existing antecedent research on ES mainly focuses on leadership style (such as abusive leadership, authoritarian leadership, and destructive leadership), personality traits (such as self-esteem, proactive personality, and regulatory focus), and other aspects (Pinder and Harlos, 2001; Duan et al., 2018; Karabay et al., 2018; Lam and Xu, 2019). However, the studies on the impact of negative events in organizational factors are still sparse. According to Jahanzeb and Fatima (2018), workplace ostracism is a source of stress which causes employees to respond with defensive silence. Similar to workplace ostracism, workplace bullying (WB) is a common and harmful event in the workplace. Therefore, the literature calls for more studies on WB (Stagg et al., 2011; Yao et al., 2020b). Rai and Agarwal (2018) echo the call with a survey of Indian employees and find that when employees are bullied in the workplace, they will think that the organization fails to fulfill its psychological contract and thereby remain silent as a passive coping strategy. However, this simple passive response of employees to a certain pressure is not sufficient to explain the complicated relationship between pressure and behavior. Instead, there exists a process of “event—cognition—affective—behaviour.” Due to the superiority of conservation of resource theory (COR) in explaining stress (Halbesleben et al., 2014), this paper attempts to explore the underlying mechanism between workplace bullying and ES based on COR.

First, COR argues that when an individual faces resource consumption, the individual tends to preserve its resources to prevent further consumption (Halbesleben et al., 2014). Since workplace bullying will consume a large number of resources, individuals have to choose silence to preserve their remaining resources. To specify, workplace bullying will result in the unsafe psychological cognition of employees (psychological safety, PS) and consume a large number of their cognitive resources (Fasanya and Dada, 2016; Yao et al., 2020a, b). After consuming cognitive resources, individuals will cherish their resources more and re-examine their value recognition to the organization (affective commitment, AC) (Yao et al., 2020b), thus adopting some self-protective behaviors (such as employee silence) to avoid the continuous consumption of emotional resources. Therefore, PS and AC are selected as mediating variables in this paper. Moreover, cognitive-affective personality system theory (CAPS) points out that when an employee encounters an event, this event will affect his/her cognitive-affective unit and thus affect his/her behavior (Mischel and Shoda, 1995). Since PS represents the self-perception of an employee of the safety degree in a certain work environment (Dontsov et al., 2018) and AC represents the emotional attachment of an individual to the organization (Vandenberghe and Bentein, 2009; Loi et al., 2012; Charbonneau and Wood, 2018), this paper constructs a chain mediating path to explore the relationship between WB and ES. Finally, previous studies find that a strong forgiveness climate (FC) in an organization can help members to ease their emotions, and thus affect their behaviors (Guchait et al., 2016, 2019; Yao et al., 2020a, b). However, research about whether FC can moderate the chain of PS and AC between workplace bullying and ES has yet to be looked into. Therefore, drawing on COR and CAPS, we deeply analyze the underlying mechanism between WB and ES to provide suggestions for organizations to reduce ES.

## Research Hypothesis

### Workplace Bullying and Employee Silence

WB refers to harassment, offense, social exclusion, or negative instances that will interrupt the work of others. It includes overt bullying such as intimidation, criticism, and humiliation, as well as covert bullying like verbal violence, excessive workload, cold violence, and unfair and disrespectful treatment (Escartin et al., 2011; Rai and Agarwal, 2018; Yao et al., 2020b). Compared with other negative behaviors such as abusive management and workplace exclusion, WB brings more harm to employees. For instance, it may reduce employees’ job satisfaction, increase their absenteeism rate and turnover intention, and damage their psychological and physical health (Glambek et al., 2014; Magee et al., 2017; Choi et al., 2018; Finstad et al., 2019). ES was first used to describe the behavior that employees use when they do not disclose their clear understanding of the organizational environment to people who can improve the situation (Morrison and Milliken, 2000; Pinder and Harlos, 2001; Knoll and Dick, 2013; Whiteside and Barclay, 2013; Prouska and Psychogios, 2018). With in-depth research, many scholars have reached a consensus that ES is a multidimensional structure, which can either be divided into acquiescent silence, defensive silence, pro-social silence, and opportunistic silence (Dyne et al., 2003), or be classified into acquiescent silence, defensive silence, and indifferent silence (Morrison and Milliken, 2000; Knoll and Dick, 2013). Due to the great harm of ES, the academic community advocates studying the reasons behind this behavior. Some studies advance this call by proposing that ES is the most common reaction after being bullied by others (Rai and Agarwal, 2018; Yao et al., 2020b). However, to the best of our knowledge, the relationship between WB and ES has not yet been explored (Rai and Agarwal, 2018; Yao et al., 2020b). Therefore, this paper attempts to extend the existing research by revealing the underlying mechanism between WB and ES. It is worth mentioning that we focus on the relationship between WB and the silent behavior of employees, without breaking down the motivation behind such behavior.

According to COR, when individuals with fewer resources face resource consumption, they are more likely to be stressed and fall into a loss spiral, thus accelerating the consumption of their resources (Hobfoll, 1989; Hobfoll et al., 2018). Therefore, individuals tend to perform behaviors that can avoid resource loss or further consumption, ensuring that there are enough resources to resist potential threats (Wheeler et al., 2010). As a common stressor, WB may lead to changes in employees’ resources, such as the continuous loss of status, dignity, time, and energy (Hobfoll, 1989; Wheeler et al., 2010; Rai and Agarwal, 2018). If employees have very few resources or their speed of resource acquisition cannot keep up with the speed of resource consumption, they may be vulnerable to a loss spiral and consume remaining resources quickly (Hobfoll, 1989; Halbesleben et al., 2014). To prevent further consumption, employees are more inclined to adopt a defensive stance to withhold remaining resources, so keeping silent is a deeply pondered decision. Although COR also points out that the individual should strive to obtain more resources rather than only avoid resource consumption (Wheeler et al., 2010; Prapanjaroensin et al., 2017; Rai and Agarwal, 2018), this will occupy the resources of individuals used for dealing with other events, which in turn hinders individuals from obtaining more resources effectively (Jin et al., 2018; Rai and Agarwal, 2018). As mentioned above, we propose the following hypothesis.

Hypothesis 1: workplace bullying is positively correlated with employee silence.

### The Mediating Effect of Psychological Safety

Psychological safety (PS) refers to a subjective cognition and feeling that an individual can freely express himself/herself at work without considering the consequences (Kahn, 1990; Lyman et al., 2020). Since such cognition is formed after long-term interpersonal interaction among employees and other organizational members, it largely depends on the respect and trust of others. Recent trends in the employee silence literature consider PS as an important cognitive variable (Duan et al., 2018; Elsaied, 2019). Adopting this perspective, we propose that PS can act as a mediator between WB and ES. First, WB can lower the self-esteem of employees. Both bullying that comes from superiors and colleagues can lead to the frustration of employees and lower self-esteem, and this type of “critical” aggression will undoubtedly reduce the PS of employees (Ouimet, 2013; Nguyen et al., 2017; Arnetz et al., 2019b). Therefore, after being bullied in the workplace, employees often choose to keep silent to prevent the harm caused by continuous resource loss. Second, WB will lower employees’ trust in other members. Existing studies have found that when employees trust organizational members more, they will have higher PS (Thirlwall, 2015; Koopmann et al., 2016; Arnetz et al., 2019a). Employees (especially new hires) who suffer WB such as being given an excessive workload, tend to highly distrust their bullies (Thirlwall, 2015; Logan and Malone, 2018). Such a low level of trust will reduce the PS of employees. As a result, even if employees find problems existing in the organization, they will choose to keep silent to avoid further loss of their resources (Hobfoll, 1989). Furthermore, WB makes it difficult for employees to get fair treatment from their leaders and colleagues. The premise of WB is an unequal relationship between the victims and bullies, including power, seniority, age, ability, status, and other aspects (Yao et al., 2020b). Due to this unequal relationship, employees are more likely to suffer frequent WB. Hence, they can neither hold different opinions with bullies nor express themselves freely due to a lack of PS (Boddy, 2011; Kang et al., 2019). In this case, in order to avoid consuming resources, employees being bullied will choose to keep silent even if they have different opinions (Duan et al., 2018; Elsaied, 2019; Yao et al., 2020b).

Hypothesis 2: psychological safety mediated the relationship between workplace bullying and employee silence.

### The Mediating Effect of Affective Commitment

Affective commitment (AC) refers to the affective attachment of individuals to other organizational members. It enables employees to maintain a semblance of alignment with the values of others and form their inner work values imperceptibly, which in turn affects their behaviors (Egri and Ralston, 2004; Vandenberghe and Bentein, 2009; Yousaf et al., 2013). As a negative event, WB may force employees to work harder, which requires employees to spend more resources such as sacrificing rest time and maintaining high concentration continuously (Rai and Agarwal, 2018; Rosander and Blomberg, 2019; Yao et al., 2020a, b). On the one side, the overload pressure brought by WB will deplete the resources of employees constantly despite their unrecovered emotional resources, and thereby lead to lower AC for preventing excessive consumption of resources (Rai and Agarwal, 2018; Tetteh et al., 2020). On the other side, failing to complete the task may cause the employee to be criticized, accused, insulted, and threatened, which will increase the loss of employees’ emotional resources, especially for employees who lack such resources. In this situation, employees are likely to fall into the loss spiral, and thus reduce their AC to withhold remaining resources after being bullied at work (Loi et al., 2012). In addition, the literature suggests that when employees’ AC is high, they will have more emotional resources and have higher levels of organizational identification and loyalty. This will encourage employees to give advice spontaneously (Wang et al., 2014; Kodama et al., 2016). On the contrary, employees with low AC will have fewer emotional resources, so they may not pay much attention to the problems existing in management decisions or in colleagues’ work. Even if they find these problems, they will choose to save resources and perform silent behaviors to prevent continuous resource consumption (Loi et al., 2012; Kodama et al., 2016; Yao et al., 2020b). Therefore, WB is likely to promote silent behavior by reducing AC.

Hypothesis 3: affective commitment mediated the relationship between workplace bullying and employee silence.

### The Chain Mediating Effect

As CAPS proposes, individual cognitive-affective units are not independent, instead, they have a mutual effect (Mischel and Shoda, 1995). To specify, when an individual encounters an event, either his/her cognition or affection will change, thus affecting his/her behavior. Or, cognitive units and affective units can influence and transform mutually, and ultimately affect behaviors (Lee and Pee, 2015). According to previous studies, PS represents a kind of cognition that individuals can freely show themselves in the organization, which belongs to the cognitive unit (Kahn, 1990; Dontsov et al., 2018). While AC is an emotional attachment to the organization and its members, which belongs to the affective unit (Egri and Ralston, 2004; Vandenberghe and Bentein, 2009; Loi et al., 2012; Charbonneau and Wood, 2018; Jiang and Johnson, 2018). After employees suffer WB, their cognitive resources will be greatly consumed. When individuals lack resources and face resource consumption, they are likely to fall into a loss spiral, which leads to lower PS. Furthermore, after excessive consumption of cognitive resources, individuals will form rational cognition to reduce the consumption of their emotional resources. Meanwhile, they will save resources to avoid further loss of individual resources. Hence, employees will worry about the consumption of resources and choose to keep silent even if they capture problems existing in the organization (Hobfoll, 1989; Eaton et al., 2009; Lapointe et al., 2011; Hobfoll et al., 2018). Thus, WB activates the affective unit (AC) by stimulating the individual’s cognitive unit (PS) and ultimately affects the behavior of the individual (employee silence).

Hypothesis 4: psychological safety and affective commitment have a chain mediating role between workplace bullying and employee silence; that is, workplace bullying can reduce the affective commitment of individuals by reducing his/her psychological safety, thereby leading to the employee silence.

### The Moderating Effect of a Forgiveness Climate

A forgiveness climate (FC) mediates the degree to which an individual performs the tolerance and kindness expected by the organization when he or she has been offended by other organizational members (Fehr and Gelfand, 2012; Yao et al., 2020a, b). When FC is high, individuals tend to believe that they will be rewarded and supported if they show tolerance and kindness toward offense (Guchait et al., 2016, 2019; Yao et al., 2020a, b). As studies propose, FC can soothe the offended people, repair damaged interpersonal relationships, and create a friendly work environment (Guchait et al., 2016, 2019). Thus, FC can guide individuals to perform behaviors that benefit the organization, and it is an environmental variable that can facilitate the fulfillment of basic personal needs (Salvador, 2020; Yao et al., 2020a, b).

CAPS points out that employees have two modes of response: the “cold” processing of rational cognition and the “hot” processing of emotional impulses (Mischel and Shoda, 1995; Lee and Pee, 2015). Although many studies suggest that “hot” emotional impulses need to be transformed into “cold” rational cognition, these two responses have no particular order. Instead, it depends more on the events encountered and individuals’ processing patterns (Mendoza-Denton et al., 1997; Kell, 2018). In other words, after receiving external stimulation, individuals will consider the influences of stimulation comprehensively. Then two reaction modes will transform to each other, and finally generate rational behaviors (Ayduk and Gyurak, 2008; Frieder et al., 2018; Kell, 2018). If a certain environmental variable can change the two reaction modes into each other under the stimulus, such a variable can effectively moderate the impact of events on employees’ cognition or emotion (Mischel and Shoda, 1995; Ayduk and Gyurak, 2008; Yao et al., 2020a, b).

In a strong FC, even if an individual experiences WB, the individual may regard it as an exercise rather than a deliberate provocation or unfair behavior. For example, when organizational members or superiors give employees excessive work, the employee (especially new hires) will perceive such “bullying” as an opportunity to show himself/herself, hence will neither cause excessive loss of cognitive resources nor reduce PS (Guchait et al., 2016, 2019; Bell and Fincham, 2019). Under such a high level of FC, organizational members tend to tolerate individuals even if they are unable to complete the task. Such tolerance can supplement the cognitive resources consumed by individuals (Lee and Pee, 2015; Guchait et al., 2019; Yao et al., 2020a). On the contrary, under a low level of FC, employees find it difficult to forgive and are more likely to preserve cognitive resources. Furthermore, FC can moderate the negative effect of WB on PS, thus influencing the chain mediating effect of PS and AC between WB and ES. At a low level of FC, employees tend to form rational cognition and turn it into an emotional impulse. This will allow employees to save enormous emotional resources, thereby increasing the possibility of employees to produce silent behavior. In contrast, in a high level of FC, employees can alleviate the consumption of cognitive resources caused by WB, thus reducing the consumption of emotional resources and supplement resources by the tolerance of organizational members. These resources can promote employees to acquire a higher sense of responsibility and reduce the possibility of silent behaviors. Therefore, strong FC can reduce the negative impact of WB on PS, and will weaken the chain of mediating effect between WB and ES. Based on the above analysis, we propose the following hypothesis.

Hypothesis 5: a forgiveness climate moderates the negative effect of workplace bullying on psychological safety, as well as moderates the indirect effect of workplace bullying on employee silence via psychological safety and affective commitment.

**FIGURE 1:**
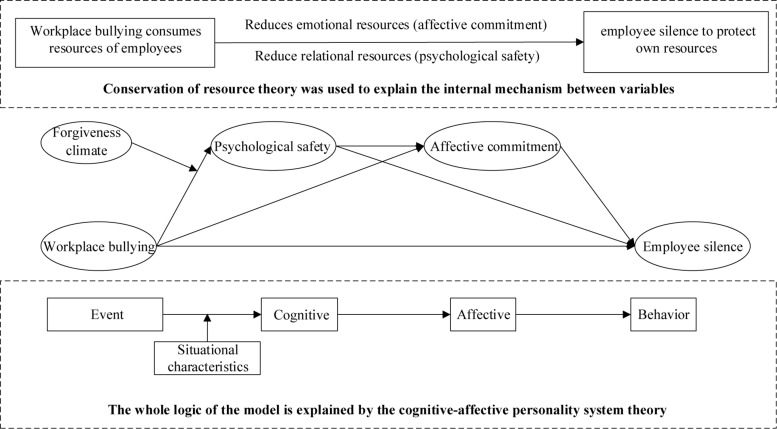
Theoretical model.

Figure 1 presents our research model.

## Materials and Methods

### Samples and Procedures

The research team contacted the leaders of three private enterprises in Shenzhen, Changsha, and Nanning in China. These enterprises are mainly engaged in information communication, machinery manufacturing, and educational business training. All of them have been established for more than 5 years and have more than 300 staff. Since one of our research group keeps close contact with the senior manager of the three enterprises and we promised to share the research results with the surveyed enterprises, we have received strong support from senior managers so that the research group can survey employees freely. In order to avoid serious common method variance (CMV) in the research results, this study conducted a questionnaire survey at two time points. In the first survey (time 1), we collected data on employees’ basic information and measured WB as well as FC. Two months later (time 2), we collected data on PS, AC, and ES.

The specific collection procedures are as follows: first, before the questionnaire was distributed, the research group stated that the survey is anonymous and the data are only used for academic research. Second, during the process of filling in the questionnaire, members of the research group will wait nearby. If participants have any questions, they can ask for help. Third, after the questionnaire is completed, we immediately collect it and put it in the envelope for anonymous numbering. Finally, participants who participated in both surveys were given a box (about $10) of toothpaste prepared by the research group. In this survey, a total of 400 questionnaires are issued. Of these, 351 were valid at time 1 and 322 at time 2 (after eliminating invalid questionnaires such as too many missed answers and irregular answers). The overall response rate is 80.05% (57.10% are males and 42.90% are females). The average age of participants is 31.888 years (SD 6.264). Regarding educational background, a bachelor’s degree or below accounts for 59.90%, postgraduate degree or above accounts for 40.10%. The average time of working in the company is 5.717 years (SD 4.814).

### Measures

Since the scales are all in English, we invited a university teacher majoring in English to carry out a strict “translation-back” procedure and invited two doctoral students to compare and check the Chinese scales as well as the English scales to avoid semantic ambiguity. We use Likert-5 to score each item (the higher the score, the more the individual’s situation corresponds to the item). The source of the scale is as follows:

***WB***. A nine-item scale developed by Einarsen et al. (2009) is used to measure WB. Sample items include “Being exposed to an unmanageable workload” and “Being ordered to do work below my level of competence,” etc.
***FC***. We used the four-item scale developed by Cox (2008) to measure FC. Sample items include “We are forgiving of each other’s offenses” and “We are able to work through our differences,” etc.
***PS***. We mainly refer to the PS scale developed by Edmondson (1999) and Detert and Burris (2007) to measure the variable, and there are 5 items in total. Sample items include “It is safe for me to speak up around here” and “No one on this organization would deliberately act in a way that undermines my efforts,” etc.
***AC***. We mainly refer to the AC scale developed by Allen and Meyer (1990), and there are 8 items in total. Sample items include “I would be very happy to spend the rest of my career with this organization” and “I enjoy discussing my organization with people outside it,” etc.
***ES***. We mainly refer to the ES scale developed by Van Dyne et al. (2003), and there are 5 items in total. Sample items include “I don’t speak up and suggest ideas for change, based on fear” and “I omit pertinent facts in order to protect myself,” etc.
***Control variables***. In this paper, gender, age, educational background, and working-age are selected as control variables. On the one side, existing studies have found that female employees are more likely to be affected by negative events in their mood and behavior (Warren and Catherine, 1999), so we used gender as a control variable. On the other side, previous studies on ES show that employee demographic variables are all important factors that can influence employee behaviors (Whiteside and Barclay, 2013; Rai and Agarwal, 2018; Srivastava et al., 2019). Therefore, gender, age, education, and the working age of employees are taken as control variables in the data analysis.

### Descriptive Statistics and Correlation Analysis

Mean and SD of control variables, WB, FC, PS, AC, and ES are shown in Table 1. Results demonstrate that WB is negatively correlated with PS (*r* = −0.240, *p* < 0.01) and AC (*r* = −0.305, *p* < 0.01) while it is positively correlated with ES (*r* = 0.513, *p* < 0.01). Moreover, PS is significantly positively correlated with AC (*r* = 0.544, *p* < 0.01), while have significantly negative correlation with ES (*r* = −0.219, *p* < 0.01). In addition, AC is significantly negatively correlated with ES (*r* = −0.228, *p* < 0.01).

**TABLE 1:** Descriptive statistics and correlation analysis of each variable.

Variables	Sex	Age	Education	Working age	WB	FC	PS	AC	ES
Sex	−								
Age	–0.066	−							
Education	–0.060	0.178**	−						
Working age	–0.017	0.383**	–0.043	−					
WB	–0.075	–0.075	–0.051	–0.062	***0.857***				
FC	0.005	0.021	–0.036	0.049	0.269**	***0.866***			
PS	0.028	0.056	0.019	0.071	−0.240**	–0.067	***0.879***		
AC	0.036	0.074	0.010	0.049	−0.305**	−0.118*	0.544**	***0.922***	
ES	–0.063	–0.021	–0.046	–0.022	0.513**	0.196**	−0.219**	−0.228**	***0.884***
Mean	0.429	31.888	0.599	5.717	3.916	3.443	2.992	2.805	3.447
SD	0.496	6.264	0.491	4.814	0.645	0.711	0.562	0.410	0.661

**p < 0.01, *p < 0.05, two-tailed test. Bold and slanted data on diagonal is variables’ Cronbach’ s Alpha. WB, FC, PS, AC, ES stand for workplace bullying, forgiveness climate, psychological safety, affective commitment and employee silence respectively, the same below. 0 = male, 1 = female; 0 = postgraduate degree or above, 1 = bachelor’s degree or below.

## Results

### Reliability and Validity

The reliability coefficient of each variable is calculated in this paper. The results are shown in Table 1. Cronbach’s Alpha values all are greater than 0.8, which indicates the good reliability of the scale. In addition, to test whether these scales are differentiated, this paper uses AMOS 22.0 software to test the validity of WB, FC, PS, AC, and ES (see Table 2). The results show that the fitting degree of the five-factor model (χ^2^/df = 2.180, IFI = 0.914, TLI = 0.904, CFI = 0.913, RSMEA = 0.061) is significantly better than other factor models, which means that these scales have good discriminative validity.

**TABLE 2:** Results of confirmatory factor analysis.

Models	χ ^2^	df	χ ^2^/df	△χ ^2^	IFI	TLI	CFI	RMSEA
WB, FC, PS, AC, ES	911.268	418	2.180	–	0.914	0.904	0.913	0.061
WB + FC, PS, AC, ES	1402.434	422	3.323	491.166***	0.829	0.810	0.828	0.085
WB + FC, PS + AC, ES	1865.164	425	4.389	953.896***	0.749	0.723	0.747	0.103
WB + FC, PS + AC + ES	2322.264	427	5.439	1410.996***	0.670	0.638	0.667	0.118
WB + FC + PS + AC + ES	3446.964	428	8.054	2535.696***	0.474	0.424	0.470	0.148

***p < 0.001. +Represents the combination of two factors into one factor.

Although our study design includes two time points to avoid CMV, it only has one source. Therefore, it is necessary to further examine the CMV problem. First, we adopt the Harman single-factor method. The results show that in the factor with a characteristic root greater than 1, the explanatory volume of overall variation is 67.979%, and the first principal component is 22.636%, which neither exceeds 50% of the critical value nor exceeds half of the explanatory volume of overall variation (Podsakoff et al., 2003). In addition, the single-factor fit indices in Table 2 (χ^2^/df = 8.054, IFI = 0.474, TLI = 0.424, CFI = 0.470, RSMEA = 0.148) are not ideal. In summary, the CMV in this study will not seriously affect the results.

### Hypothesis Testing

To test and verify the theoretical model of the research, this paper builds a basic model (no direct effect of WB on ES), a nested model (on the basis of the basic model with the direct effect of WB on ES), and an alternative model (no mediating effect, WB, forgiveness climate, PS, and AC directly affect ES) to find the most optimal model.

First, we compare the fit indices of the basic model with that of the nested model. The result shows that the fit indices of the nested model (χ^2^ = 702.525, df = 323, χ^2^/df = 2.175, IFI = 0.944, TLI = 0.926, CFI = 0.938, RMSEA = 0.052) are more ideal than that of the basic model (χ^2^ = 822.636, df = 324, χ^2^/df = 2.539, IFI = 0.886, TLI = 0.875, CFI = 0.896, RMSEA = 0.064). In other words, the model’s fitting degree will improve significantly if a direct path is added. Therefore, the nested model is superior to the basic model. Similarly, we compare the fit indices of the nested model with that of the alternative model. Again, the fit indices of the nested model are better than that of the alternative model (χ^2^ = 1785.001, df = 431, χ^2^/df = 4.142, IFI = 0.764, TLI = 0.744, CFI = 0.762, RMSEA = 0.099). Therefore, this paper chooses the nested model for hypothesis testing.

#### Mediating Effect Test

**FIGURE 2:**
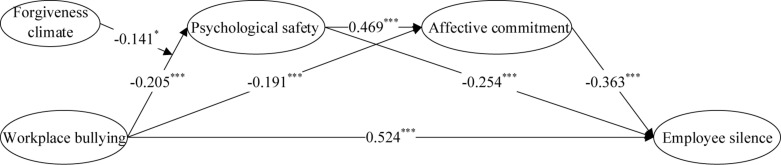
Mediating-moderating model estimation. In order to keep the graphics concise, this paper does not draw the path coefficients of control variables to core variables into the graph; The results in the figure are obtained from two analyses: mediating model test results and mediating effects of workplace bullying and forgiveness climate on the first stage;+ was significantly correlated at the level of 0.1 (bilateral), and *** was significantly correlated at the level of 0.001 (bilateral).

We use the Mplus7.4 software to verify the hypothesis, the mediation effect test is carried out by bootstrapping (see Figure 2 and Table 3).

**TABLE 3:** Bootstrapping mediation effect test.

**Path**	**Indirect effect estimation**	**Confidence interval of bia-corrected 95%**	
		**Lower**	**Upper**
Total indirect effect	0.289	0.002	0.620
**Specific indirect effect decomposition**			
WB → PS → ES	0.216	0.044	0.581
WB → AC → ES	0.038	0.147	0.266
WB → PS → AC → ES	0.035	0.148	0.221

*n = 322, Bootstrapping randomly sampled 5,000 times.*

First, the path coefficient of WB to ES is 0.524 (*p* < 0.001), indicating that WB has a significant positive impact on ES, and hypothesis 1 is supported.

Second, the path coefficient of WB to PS is −0.205 (*p* < 0.001), which indicates that there is a significant negative relationship between WB and PS. Meanwhile, the path coefficient of PS to ES is −0.254 (*p* < 0.001), which means that there is a significant negative relationship between these two variables. Since hypothesis 1 is supported, we believe that PS has a significant mediating effect in the link of WB and ES (*b* = 0.216, bootstrapping = 5,000, 95% confidence interval is [0.044, 0.581], excluding 0), and hypothesis 2 is supported.

Third, the path coefficient of WB to AC is −0.191 (*p* < 0.001), which demonstrates that WB has a significant negative relationship with AC. The path coefficient of AC to ES is −0.363 (*p* < 0.01), indicating that there is a significant negative relationship between AC and ES. Since hypothesis 1 is supported, we believe that AC has a significant mediating effect in the link of WB and ES (*b* = 0.038, bootstrapping = 5,000, 95% confidence interval is [0.147, 0.2661], excluding 0), and hypothesis 3 is supported.

Finally, the path coefficient of PS to AC is 0.469 (*p* < 0.001), which indicates that PS has a significant positive effect on AC, that is, the higher the level of PS, the higher the individual’s AC. In light of the above, this paper proposes that there is a significant chaining mediating effect of PS and AC on the relationship between WB and ES (*b* = 0.035, bootstrapping = 5,000, 95% confidence interval is [0.148, 0.221], excluding 0), and hypothesis 4 is supported.

#### Moderating Effect Test

**FIGURE 3:**
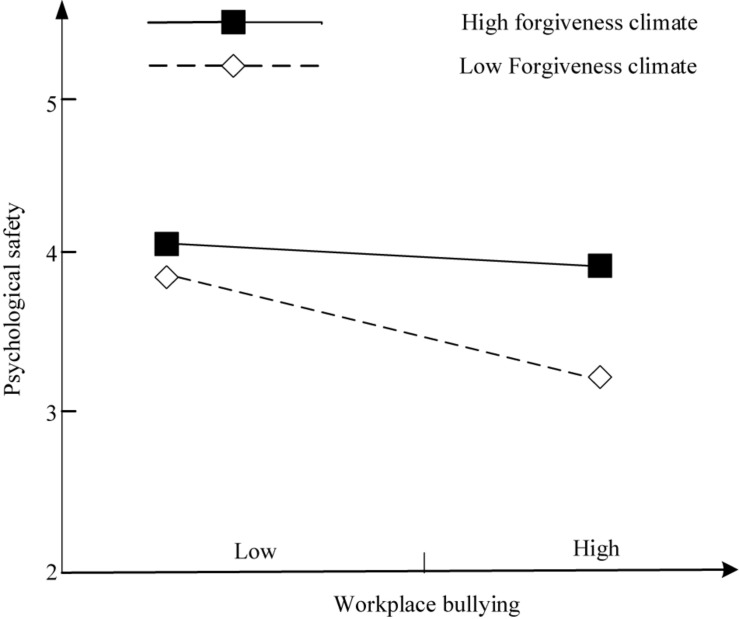
The moderating effect of the forgiveness climate.

We use a simple slope test to exam the moderating effect. The results show that WB has no significant effect on PS (*b* = −0.102, *t* = −1.208, *p* = 0.228) when the FC is high (Mean + 1 SD), while WB has a significant negative impact on PS (*b* = −0.302, *t* = −1.912, *p* < 0.1) when the FC is low (mean − 1 SD). As shown in Figure 3, the negative effect of WB on PS is more pronounced in the lower FC.

#### Moderated Chain Mediating Effect Test

We use the bootstrapping method to test the moderated chain mediating effect (Edwards and Lambert, 2007; Hayes, 2013, 2018; Hollon, 2019), and the test results are shown in Table 4. The mediating effect value is 0.021 ([0.004, 0.052]) under the high FC (mean + 1 SD) and is 0.033 ([0.007, 0.071] under the low FC (mean − 1 SD), both values reflect a significant chain mediating effect. Moreover, there are also significant differences between the chain mediating path’s indirect effect values in high FC and in low FC (*b* = −0.012, [−0.046, −0.016]). Therefore, through weakening the negative effect of WB on PS, the FC can moderate the chain mediating effect of PS and AC on the relationship between WB and ES. Hypothesis 5 is supported.

**TABLE 4:** Moderated chain mediation effect analysis.

**Moderator**	**Path: WB → PS → AC → ES**		
	**Indirect effect**	**LLCI**	**ULCI**
High FC (Mean + 1 SD)	0.021	0.004	0.052
Low FC (Mean − 1 SD)	0.033	0.007	0.071
Discrepancy	–0.012	–0.046	–0.016

## Discussion

As a negative event, workplace bullying is prevalent in the workplace. While WB is a passive negative interpersonal interaction that can damage individual resources, ES is an active negative interpersonal interaction that is used for protecting resources. Therefore, starting from the consumed resources of individuals caused by WB, this study establishes a moderated chain mediating model based on COR and CAPS. Our study finds that: (1) WB is positively correlated with ES; (2) PS and AC play a mediating role between WB and ES, respectively, and these two variables have a chain mediating effect in the above relationship; and (3) the FC moderates this chain mediating effect by buffering the negative impact of WB on PS. These results are obtained through strict questionnaire survey procedures and appropriate statistical methods, and they have good external validity. First, our results are statistically significant. Second, we conducted a large number of surveys on employees to make the research results reliable in management practice. Third, as a negative workplace event, WB will bring a series of negative effects on employees, and employees will experience the process of consuming cognitive and emotional resources. In this process, some organizational situation factors may influence the effects of WB on employees. This shows that our research has a good theoretical basis.

### Theoretical Implications

Our research conclusion has some theoretical contributions. First, from the perspective of resource gain and loss, this paper considers the behavioral response of employees after they suffer negative workplace events in the Chinese context. By exploring the influence of WB on ES, this study is helpful to supplement the antecedent variable of ES in the Chinese context, so as to increase academic attention to WB. Since China is a collectivist country with high power distance, WB should be more common in this context than in western countries (Yao et al., 2020b). To the best of our knowledge, this paper is the first empirical study to link WB to ES in the Chinese context, which responds to the call of Xu et al. (2015) for studies on the relationship between negative events and ES in a different context. Moreover, in the Chinese context, employees are more likely to follow the golden mean and remain silent due to the influence of the Confucian culture. Meanwhile, the research conclusions of this paper also confirm the finding of Rai and Agarwal (2018) in the Indian context. Also, while COR has been widely used in stress-related research, its application to WB is scant (Glambek et al., 2014; Yao et al., 2020b). Therefore, this paper enriches the literature by providing a new perspective to explore the relationship between negative workplace events and individual behavior.

Second, with the help of CAPS, we construct a chain mediator model for psychological security and affective commitment of the indirect effect of workplace bullying on employee silence. Our paper explores the transmission mechanism between WB and ES, which not only broadens the application scope of CAPS, but also avoids a drawback that exists in the study of Rai and Agarwal (2018) that is limited to the psychological contract between individuals and organizations. Due to the complexity of humanity, it is often difficult to clarify the mediation mechanism between negative events and individual behaviors in the workplace through a single variable (Yao et al., 2020a, b). This transmission will go through a whole process from “event—cognition—affective—behavior” (Mischel and Shoda, 1995; Ayduk and Gyurak, 2008; Lee and Pee, 2015; Frieder et al., 2018; Kell, 2018). This paper proves that PS and AC play a separate mediating role between WB and ES, and further proves that PS and AC play a chain mediating role between them. Therefore, this paper strengthens scholars’ understanding by explaining of the internal mechanism between WB and ES.

Finally, this paper finds an important boundary condition in the impact of WB on ES. According to our findings, we suggest that the interaction between events and the environment can influence the cognitive-affective unit. In a high FC, employees will try to adjust themselves even if they are bullied (Mischel and Shoda, 1995; Guchait et al., 2016, 2019). To specify, they will try their best to make themselves undergo “cold” processing to form rational cognition, and then convert to the “hot” processing system of emotional impulse, so as to alleviate the negative effects brought by WB. In sum, our study complements the CAPS research and helps to understand the differences as well as relationships among the constructs embodied in the theory. We also identify a boundary condition for the impact of WB on ES (Mischel and Shoda, 1995; Lee and Pee, 2015; Kell, 2018; Yao et al., 2020b). This exactly echoes the call of many scholars that we need to pay more attention to the important role of situational characteristics in the relationship between negative workplace events and employees’ emotions or behaviors (Yao et al., 2020b).

### Practical Implications

First, organizations should take active measures to curb WB. For example, organizations should itemize the bullying behaviors that have been found and develop relevant punitive policies against bullies to eliminate bullying in the workplace. Moreover, organizations should regularly carry out relevant training to make victims realize that bullying behavior is strictly prohibited by the organization, and encourage them to actively report such behavior with an “anonymous letter” or an “anonymous call.”

Second, organizations should promptly supplement the individual’s cognitive and emotional resources (such as improving individual psychological safety and affective commitment). For instance, the organization needs to implement the people-oriented management mode, care about the real thoughts of individuals, and give employees some opportunities to show themselves. In addition, the organization should advocate harmonious and correct values, and only evaluate organizational members by “what they do” instead of “who they are,” so as to improve the psychological security of individuals. Moreover, the organization should take more care of employees and help them replenish the consumed emotional resources in time. Leaders should encourage employees when they fail to complete tasks, in a timely manner. Meanwhile, organizations should emphasize cooperation when they arrange the work, such teamwork is conducive to the formation of a good interpersonal relationship among organizational members. Furthermore, the organization can provide a place such as an emotional venting room for employees to vent their emotions, which can help them to form a strong emotional attachment to the organization.

Finally, the organization should pay attention to the working atmosphere. For example, an organization can establish a tolerant organizational culture and provide employees with a forgiving climate of mutual tolerance, mutual forgiveness, friendship, and mutual assistance. Moreover, some studies have found that the fault-tolerant behavior of leaders can increase the enthusiasm of employees toward voice behavior (Yao et al., 2020c). Based on the conclusions of this paper, organizations should establish corresponding systems such as a fault tolerance mechanism and a voice encouragement mechanism.

### Limitations and Future Research Directions

Although this paper puts forward some novel ideas, there are still some limitations due to our ability and objective factors. First, all scales are from English journals. Although strict translation-back procedures are used to reduce errors, direct application to the Chinese context may have some limitations. Second, all the items of the questionnaire were filled in by the same employee, which may inevitably lead to CMV. Although this paper has been circumvented in procedure and method, the subsequent research should eliminate it by controlling the survey procedure combined with multiple data collection sources (Malhotra et al., 2006; Spector, 2006). Third, the fit indices effect of our confirmatory factor analysis is not very good. In future studies, we will take some measures (such as increasing the sample size and improving the quality of the questionnaire) to improve the fit indices. Fourth, based on CAPS, we explore the inner mechanism between WB and ES, and demonstrate the important role of emotional and cognitive resources in that relationship. However, cognitive resources and emotional resources include many other variables, such as interpersonal trust and relationship identification, organizational identification, and psychological distress (Fulmer and Gelfand, 2012; Qu et al., 2015; Shen et al., 2019; Yao et al., 2020a, b), which need to be verified by empirical investigation in the future. In addition, we only analyze the mediating variables that may exist in the above relationships at the individual level. There may also be mediating variables that we have not yet discovered at the team level and organizational level. In the follow-up study, new variables can be found from these levels. Fifth, other boundary conditions are yet to be discovered by scholars. This paper only verifies that a forgiveness climate can moderate the negative effects of WB. At the beginning of the research design, this paper also considered empowering leadership and ethical leadership as boundary conditions, but the results could not be confirmed. In future studies, other situational characteristics (such as psychosocial safety climate), leadership styles, and organizational factors (such as work-related stress) are also likely to serve as boundary conditions between WB and employees’ emotions or behaviors (Vignoli et al., 2015; Dollard et al., 2017), which still need to be further explored.

## Data Availability Statement

The raw data supporting the conclusions of this article will be made available by the authors, without undue reservation, to any qualified researcher.

## Ethics Statement

The studies involving human participants were reviewed and approved by the Academic Ethics Supervision Research Institute of Hunan University. The patients/participants provided their written informed consent to participate in this study.

## Author Contributions

XL conceived and designed the study, and completed the manuscript in English. ZY participated in drafting the manuscript and revised it critically for critical intellectual content. SY gave many good research advices and revised the manuscript. All authors contributed to the article and approved the submitted version.

## Conflict of Interest

The authors declare that the research was conducted in the absence of any commercial or financial relationships that could be construed as a potential conflict of interest.
